# M1 Macrophage-Derived Exosome LncRNA PVT1 Promotes Inflammation and Pyroptosis of Vascular Smooth Muscle Cells in Abdominal Aortic Aneurysm by Inhibiting miR-186-5p and Regulating HMGB1

**DOI:** 10.1007/s12012-024-09838-5

**Published:** 2024-03-07

**Authors:** Jinhui Zhang, Xili Zhang, Xunqiang Liu, Huanjun Chen, Jifeng Wang, Min Ji

**Affiliations:** 1 Yan’an Hospital Affiliated To Kunming Medical University, Kunming, 650032 China; 2 First Affiliated Hospital of Kunming Medical University, Kunming, 650032 China

**Keywords:** Exosome, Pyroptosis, VSMCs, Abdominal aortic aneurysm, LncRNA PVT1, miR-186-5p

## Abstract

Abdominal aortic aneurysm (AAA) is a chronic vascular degenerative disease. Vascular smooth muscle cells (VSMCs) are essential for maintaining the integrity of healthy blood vessels. Macrophages play an important role in the inflammatory process of AAA. However, the effect of macrophage-derived exosome LncRNA PVT1 on VSMCs is unclear. Exosomes from M1 macrophages (M1φ-exos) were isolated and identified. The expression of LncRNA PVT1 in M1φ-exos was determined. AAA cell model was constructed by treating VSMCs with Ang-II. AAA cell model was treated with M1φ exosomes transfected with si-LncRNA PVT1 (M1φ^si–LncRNA PVT1^-exo). VSMCs were transfected with miR-186-5p mimic and oe-HMGB1. Cell viability was detected by CCK-8. The accumulation of LDH was detected by ELISA. Western blot was used to detect the expression of HMGB1, inflammatory factors (IL-6, TNF-α and IL-1β) and pyroptosis-related proteins (GSDMD, N-GSDMD, ASC, NLRP3, Caspase-1 and Cleaved-Capase-1). Cell pyroptosis rate was detected by flow cytometry. At the same time, the targeting relationship between miR-186-5p and LncRNA PVT1 and HMGB1 was verified by double fluorescein experiment. Exosomes from M1φ were successfully extracted. The expression of LncRNA PVT1 in M1φ-exos was significantly increased. M1φ-exo promotes inflammation and pyroptosis of VSMCs. M1φ^si−LncRNA PVT1^-exos inhibited the inflammation and pyroptosis of VSMCs. LncRNA PVT1 can sponge miR-186-5p mimic to regulate HMGB1 expression. MiR-186-5p mimic further inhibited inflammation and pyroptosis induced by M1φ^si−LncRNA PVT1^-exos. However, oe-HMGB1 could inhibit the reversal effect of miR-186-5p mimic. LncRNA PVT1 in exosomes secreted by M1φ can regulate HMGB1 by acting as ceRNA on sponge miR-186-5p, thereby promoting cell inflammatory and pyroptosis and accelerating AAA progression.

**Supplementary Information:**

The online version contains supplementary material available at 10.1007/s12012-024-09838-5.

## Introduction

Abdominal aortic aneurysm (AAA) is a pathological expansion of the abdominal aorta with a diameter of > 3 cm [1]. The prevalence of AAA increases with age, and it has become one of the important diseases that threaten the life and health of our people [2]. It is reported that about 4500 people die from AAA rupture every year, and the total mortality rate is about 90% [3]. Pathologically, the occurrence of AAA is due to the loss of vascular smooth muscle cells and the degradation of extracellular matrix, resulting in changes in aortic structure [4]. At present, there is no approved drug treatment for AAA. Vascular smooth muscle cells (VSMCs) are essential for maintaining the integrity of healthy blood vessels [5]. Therefore, studying the mechanism of VSMCs loss is crucial for finding drugs for the treatment of AAA.

Macrophages are an immune cell that mainly includes M1 and M2 phenotypes. It plays a crucial role in the initial inflammatory phase of AAA [6]. The initial inflammatory phase induces stromal degeneration and VSMC apoptosis, which promotes the development of AAA [7, 8]. M1 macrophages play a pro-inflammatory role in AAA and induce matrix degeneration, while M2 cells promote the regression of inflammation and alleviate tissue remodeling [9, 10]. Exosomes are thought to be produced by all cells and exist in biological fluids. Exosomes are vesicles produced by direct budding of the plasma membrane, with a diameter of 40–160 nm [11]. Exosomes secreted by macrophages can affect multiple physiological processes of cells to regulate the occurrence and development of diseases [12, 13].They mainly achieve intercellular communication by transporting various substances such as lncRNA, miRNA, mRNA and protein [14]. For example, Nicotine-stimulated macrophage exosomes accelerate atherosclerosis through miR-21-3p/PTEN-mediated VSMC migration and proliferation [15]. M1-type macrophage exosomes are designed to promote M1 polarization and target IL-4 receptors, and inhibit tumor growth by reprogramming tumor-associated macrophages into M1-like macrophages [16]. At the same time, exosomes from M1-polarized macrophages enhance the anti-tumor activity of paclitaxel by activating macrophage-mediated inflammation [17]. However, the effect of M1 macrophage-derived exosomes on endothelial cell pyroptosis in abdominal aortic atherosclerosis is unclear.

Long non-coding RNAs (lncRNAs) constitute a class of non-coding RNAs, which are generally longer than 200 bases. These RNA molecules have been linked to the regulation of tumor growth and metastasis [18]. Notably, the lncRNA PVT1 is widely recognized as a key oncogenic factor, especially in promoting malignant behaviors in various types of tumors. Of particular interest, studies have demonstrated that the expression of LncRNA PVT1 is positively correlated with the progression of abdominal aortic aneurysm (AAA) [19, 20]. The researchers found that elevated expression of long non-coding RNA PVT1 enhanced apoptosis of vascular smooth muscle cells (VSMC) and destruction of extracellular matrix (ECM) in mouse models of AAA [21]. In addition, curcumin nicotinate has been shown to exert its anti-AAA effect by targeting the lncRNA PVT1/miR-26a/KLF4 axis [20]. However, the specific role of macrophage-derived exosomal LncRNA PVT1 in AAA progression remains unclear.

In this study, we first established an in vitro cell model of AAA by treating VSMCs with angiotensin II (Ang-II). We studied the effects of M1φ macrophage-derived exosomes on pyroptosis and inflammatory injury of AAA cells. We found that M1φ macrophage-derived exosome LncRNA PVT1-mediated miR-186-5p/HMGB1 signaling pathway promotes VSMCs pyroptosis and inflammation.

## Methods

### Cell Culture

HA-VSMCs(ATCC, USA) was cultured in DMEM supplemented with 10% fetal bovine serum (10% FBS), l-glutamine, 4-(2-hydroxyethyl)-1-piperazineethanesulfonic acid (HEPES), and pyridoxine HCl (Sigma-Aldrich, MO, USA).

Human monocyte line THP-1 cells purchased from ATCC (Rockville, USA) were seeded in 24-well plates (3 × 10^5^ per well). THP-1 cells were cultured in RPMI 1640 medium supplemented with 40 ng/mL phorbol 12-myristate 13-acetate (PMA) (Sigma-Aldrich, USA) for 24 h to differentiate into adherent macrophages. Then, RPMI 1640 medium containing 10% FBS was used to replace the medium containing PMA for 24 h. Finally, macrophages were incubated with 100 ng/mL LPS (Sigma, USA) and 20 ng/mL IFN-γ (PeproTech, Shanghai) to promote macrophage polarization to M1 type (M1φ). After 24 h of polarization, cells were collected for subsequent experiments.

### Cell Transfection

VSMCs were seeded in six-well plates (1 × 10^5^ per well) and cultured for 24 h. When the cell density reached 70%, VSMCs were transfected with lentiviral vectors containing overexpression of HMGB1 (oe-HMGB1) (Hanbio, Shanghai). VSMCs were transfected with miR-186-5p mimics and NC-mimics by Lipofectamine 2000 (Invitrogen, California, USA). Cells were collected 48 h after transfection for RT-qPCR to determine transfection efficiency. After successful transfection, VSMCs were treated with 1 μmol/L Ang-II for 24 h.

M1 macrophages were seeded in six-well plates (2 × 10^5^ per well) and cultured for 24 h. When the cell density reached 70%, the si-RNA negative control (si-NC) and the si-RNA targeting LncRNA PVT1(si-LncRNA-PVT1) were integrated into the pLenR-GPH lentiviral vector. Then 8μL lentivirus was transfected into M1 macrophages using LipofiterTM (Hanbio, Shanghai). After 48 h of transfection, the cells were collected for RT-qPCR to detect the transfection efficiency. After successful transfection, it was used for subsequent experiments. The primer sequence is as follows: si-LncRNA PVT1, Forward: 5′-CCUGAUGGAUUUACAGUGATT-3′, Reverse: 5′-UCACUGUAAAUCCAUCAGGTT-3′; si-NC: Forward: 5′-GCUACGAUCUGCCUAAGAUTT-3′, Reverse: 5′-AAUCCAUGAGGCAUUCAGCTT-3′. It was integrated into the pLenR-GPH lentiviral vector. Then 8μL lentivirus was transfected into M1 macrophages using LipofiterTM (Hanbio,Shanghai). Cells were collected 48 h after transfection for RT-qPCR to determine transfection efficiency. After successful transfection, it was used for subsequent experiments.

### Experimental Design

This experiment was mainly divided into 11 groups, which were NC group, Ang-II group, miR-186-5p mimic group, oe-HMGB1 group, M1φ-Exos group, M1φsi-NC-Exos group, M1φ^si−LncRNA PVT1^-Exos group, NC-mimic + M1φ^si−LncRNA PVT1^- Exos group, miR-186-5p mimic + M1φ^si−NC^-Exos group and miR-186-5p mimic + M1φ^si−NC^ -Exos group and miR-186-5p mimic + M1φ^si−LncRNA PVT1^-Exos group. NC group:VSMCs were cultured normally without other treatments.Ang-II group:1 μmol/L Ang-II for 24 h was used. MiR-186–5 p mimics group: miR-186–5 p mimics and 1 μmol/L Ang-II were used for 48 h; oe-HMGB1 group: The cells were treated with oe-HMGB1 and 1 μmol/L Ang-II for 48 h. M1φ-Exos group: treatment of VSMCs with 40 μg/mL M1φ-Exos and 1 μmol/L Ang-II for 24 h. M1φ-Exos group: treatment with 40 μg/mL M1φ-Exos and 1 μmol/L Ang-II for 24 h. M1φ^si−NC^-Exos group: treated with 40 μg/mL M1φ^si−NC^-Exos and 1 μmol/L Ang-II for 24 h. NC-mimic + M1φ^si−LncRNA PVT1^-Exos group: treated with NC-mimic for 48 h and then 40 μg/mL M1φ^si−LncRNA PVT1^-Exos and 1 μmol/L Ang-II for 24 h. miR-186-5p mimic + M1φ^si−NC^-Exos group: treated with miR-186-5p mimic for 48 h then 40 μg/mL M1φ^si−NC^-Exos and 1 μmol/L Ang-II for 24 h. miR-186-5p mimic + M1φ^si−LncRNA PVT1^-Exos group: treated with NC-mimic for 48 h then 40 μg/mL M1φ^si−LncRNA PVT1^-Exos and 1 μmol/L Ang-II for 24 h. miR-186-5p mimic + M1φ^si−LncRNA PVT1^-Exos group: treated with miR-186-5p mimic for 48 h then 40 μg/mL M1φ^si−NC^-Exos and 1 μmol/L Ang-II for 24 h.

### Isolation of Macrophage-Derived Exosomes

Cell supernatant was collected by ultracentrifugation. The cell debris was removed by centrifugation, and the supernatant was centrifuged again. The liquid at the bottom of the tube was collected, and PBS was added to re-suspend, and then the precipitate obtained by centrifugation was the exosome (M1φ-Exos). The precipitate was re-suspended in PBS and stored at − 80 °C for subsequent experiments.

### Identification of Exosomes

The morphology of exosomes was observed by transmission electron microscopy (TEM, Thermo Scientific, MA). Nanoparticle tracking analysis (NTA, Brookhaven, New York) measured exosome diameter and particle number. Exosome markers CD9, CD63 and tumor susceptibility gene 101 (TSG101) were detected by Western blot analysis.

### The Uptake of Exosomes

PKH-26 cells were incubated with 10 μg M1φ-Exos for 15 min at room temperature. Then 5% bovine serum albumin was added to terminate the labeling. After centrifugation and co-culture with VSMCs for 24 h, they φwere observed and photographed under confocal fluorescence microscope.

### CCK-8

VSMCs cells in each experimental group were taken. According to the manufacturer's instructions, cell viability was measured using a CCK-8 analysis kit. The absorbance was measured at 450 nm with a microplate reader.

### Detection of Lactate Dehydrogenase (LDH) Level

The LDH release level of VSMCs cells was measured by LDH assay kit (Abcam, USA). The absorbance was measured by a microplate reader at a wavelength of 490 nm, and the LDH content in the culture medium was calculated by a parallel standard curve.

### Dual-Luciferase Reporter Assay

The target binding sites of Lnc RNA-PVT1 with miR-186-5p and miR-186-5p with HMGB1 were predicted by bioinformatics database. Wild-type and mutant sequences (LncRNA PVT1-WT, LncRNA PVT1-MUT, HMGB1-WT, and HMGB1-MUT) were designed and cloned into pGL3-Promoter plasmid vector. Then, HEK293T cells were inoculated in 12-well plates. When the cell confluence reached 70%, the cells were incubated with the vectors LncRNA PVT1-WT, LncRNA PVT1 -MUT, HMGB1-WT, and HMGB1-MUT, respectively, and then incubated with the miR-186-5p mimic (30 nM, RiboBio, China) or the negative control (NC-mimic) (30 nM, RiboBio, China) for 48 h of co-transfection. Finally, a dual luciferase reporter gene assay system was used to determine luciferase activity (Promega, Madison, USA).

### Western Blotting

VSMCs in the logarithmic phase were collected, and the total protein in the cells was extracted by RAPI lysate to detect the protein concentration. The protein was isolated by 10% SDS-PAGE and the target band was transferred to PVDF membrane by electroporation. After blocking with 5% skim milk powder at room temperature for 1 h, primary antibodies were added, including HMGB1(ab18256,1:5000, Abcam) and GSDMD (ab219800,1:1000, Abcam), N-GSDMD (ab215203,1:5000, Abcam), ASC(ab151700,1:1000, Abcam), NLRP3 (ab263899,1:1000, Abcam), Caspase-1(ab138483,1:5000, Abcam), Cleaved-Caspase-1(#404991:1000, SAB), IL-6 (21865-1-AP,1:1000, proteintech), TNF-α (17590-1-AP,1:1000, proteintech), IL-1β (16806-1-AP,1:5000, proteintech) and GAPDH (60004-1-IG,1:1000, proteintech) were incubated overnight at 4 °C. After that, the HRP-conjugated Affinipure Goat Anti-Mouse IgG (SA00001-1, 1:50000, proteintech) or HRP-conjugated Affinipure Goat Anti-Rabbit IgG (SA00001-2, 1:1000, proteintech) was added and incubated at room temperature for 2 h. Finally, ECL staining was performed and images were collected by a gel imager.

### Flow Cytometry for Active Caspase-1

After trypsin digestion, the cells were collected, re-suspended in PBS solution, and subsequently counted. The cells were then labeled with Annexin V (AV) and Propidium Iodide (PI) for a brief period, before quantitative determination of pyroptosis cells using a flow cytometry instrument (guava easyCyte TM 8, Millipoll, USA).

### Reverse Transcription-Quantitative Polymerase Chain Reaction (RT-qPCR) Analysis

Total RNA of cells were extracted from the cells by RNA Fast Extraction Kit (BioTeke, Beijing, China), and the concentration of RNA was detected by NanoDrop. Subsequently, RNA was reverse transcribed into cDNA by one-step reverse transcription kit (DBI®Bioscience, Ludwigshafen, Germany), and the expression levels of LncRNA PVT1 and miR-186-5p were detected in strict accordance with the instructions of qPCR kit, with U6 as internal reference. The primer sequence is as follows: LncRNA PVT1, Forward: 5′-CATCCGGCGCTCTCAGCT-3′, Reverse: 5′-TCATGATGGCTGTATGTGCCA-3′; GAPDH Forward: 5′- GGTTGTCTCCTGCGACTTCA-3′, Reverse: 5′- TGGTCCAGGGTTTCTTACTCC-3′. According to the Ct value, the copy number was calculated by 2^−ΔΔCt^, and GAPDH was used as the internal reference.

### Statistical Analysis

The data are expressed as mean ± standard error based on at least three independent experiments. Student-t test was used for comparison between the two groups, and one-way analysis of variance (ANOVA) was used for statistical analysis in three or more groups. P < 0.05 was considered statistically significant.

## Results

### Isolation and Identification of Exosomes Derived from M1φ

**Fig. 1:**
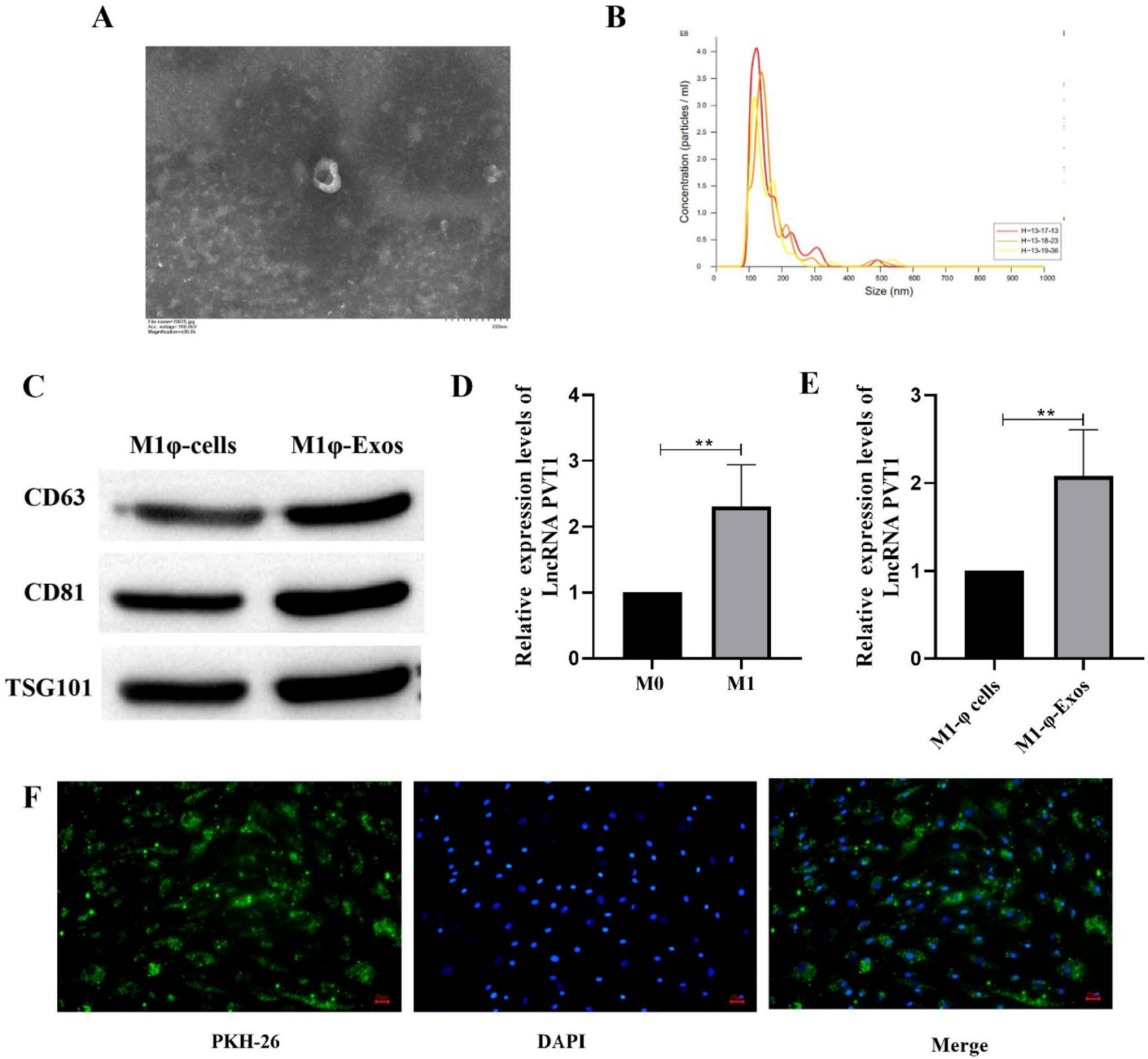
Isolation and identification of exosomes derived from M1φ. **A** Transmission electron microscope was used to detect exosome morphology; **B** NTA was used to detect the size of exosomes; **C** The expression of CD63, CD81 and TSG101 was detected by Western blot. **D** and **E** The expression of LncRNA PVT1 was detected by RT-qPCR. **F** PKH-26 was used to detect exosome intake. Each group had 3 biological replicates.*P < 0.05, **P < 0.01

In order to isolate and extract exosomes from M1φ, we induced the transformation of HTP-1 to M1φ (Fig. S1). Exosomes were extracted from M1φ, and the morphology of exosomes was observed by transmission electron microscopy. It was found that the morphology of M1φ-Exos was intact and spherical (Fig. 1A). NTA detection showed that the particle size of exosomes was between 100 and 200 nm (Fig. 1B). Western blot was used to detect exosome markers (CD63, CD81 and TSG101). The results showed that the expression of CD63, CD81 and TSG101 in M1φ-Exos was significantly increased (Fig. 1C). The expression of LncRNA PVT1 was detected by RT-qPCR. The results showed that the expression of LncRNA PVT1 in M1 macrophages was significantly increased compared with M0 cells (Fig. 1D), and the expression of LncRNA PVT1 in M1φ-Exos was also significantly higher than that in M1φ-cells (Fig. 1E). Finally, PKH-26 was used to detect the entry of M1φ-Exos into VSMCs (Fig. 1F).

### M1φ-Exos Promotes VSMCs Inflammation

**Fig. 2:**
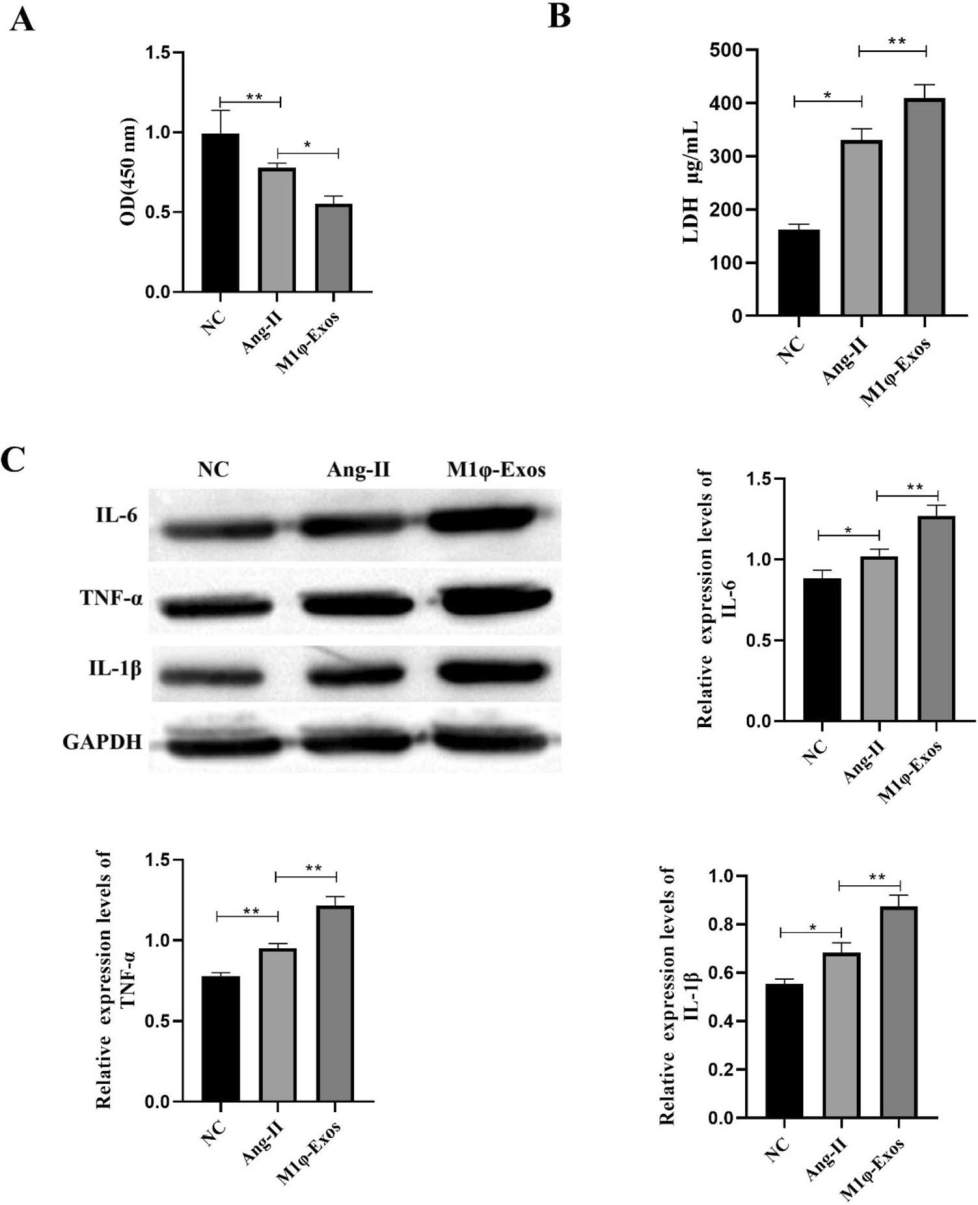
M1φ-Exos promotes VSMCs inflammation. A CCK-8 was used to detect cell viability; B ELISA was used to detect the concentration of LDH;C Western blot was used to detect inflammatory factors (IL-6, TNF-α and IL-1β). Each group had 3 biological replicates. A one-way ANOVA was performed to compare data between groups.*P < 0.05, **P < 0.01

VSMCs were treated with 100 nM Ang-II for 24 h, and cell viability and LDH concentration were measured. The results showed that compared with the NC group, Ang-II significantly inhibited cell viability and increased LDH concentration. After M1φ-Exos treatment, cell viability was further inhibited (Fig. 2A), and the concentration of LDH was further increased (Fig. 2B). The expression of pro-inflammatory factors (IL-6, TNF-α and IL-1β) was detected by Western blot. The expression of IL-6, TNF-α and IL-1β was significantly increased after Ang-II and M1φ-Exos treatment (Fig. 2C). In summary, M1φ-Exos promotes VSMCs inflammation.

### M1φ-Exos Promotes VSMCs Pyroptosis

**Fig. 3:**
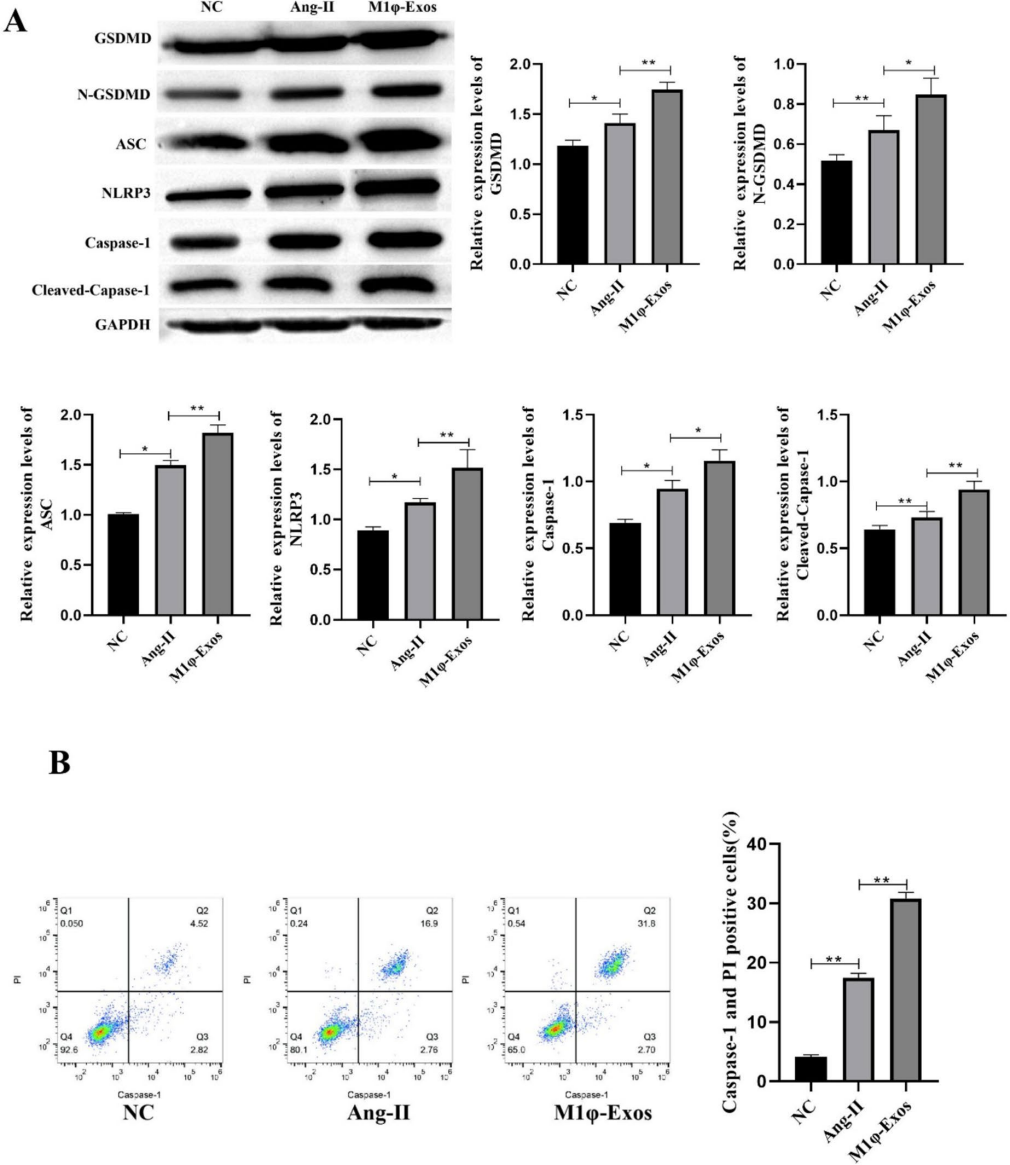
M1φ-Exos promotes VSMCs pyroptosis. A Western blot was used to detect pyroptosis-related proteins; B pyroptosis was detected by flow cytometry. Each group had 3 biological replicates. A one-way ANOVA was performed to compare data between groups.*P < 0.05, **P < 0.01

In order to explore the effect of M1φ-Exos on pyroptosis of VSMCs. Pyroptosis was detected by Western blotting and flow cytometry, respectively. Western blot results showed that Ang-II could significantly promote the expression of GSDMD, N-GSDMD ASC, NLRP3, Caspase-1 and Cleaved-Caspase-1. M1φ-Exos treatment further promoted their expression (Fig. 3A). Flow cytometry detection of pyroptosis also obtained similar results (Fig. 3B). In summary, M1φ-Exos can promote VSMCs pyroptosis.

### LncRNA PVT1 Derived from M1φ-Exos Promotes VSMCs Inflammation

**Fig. 4:**
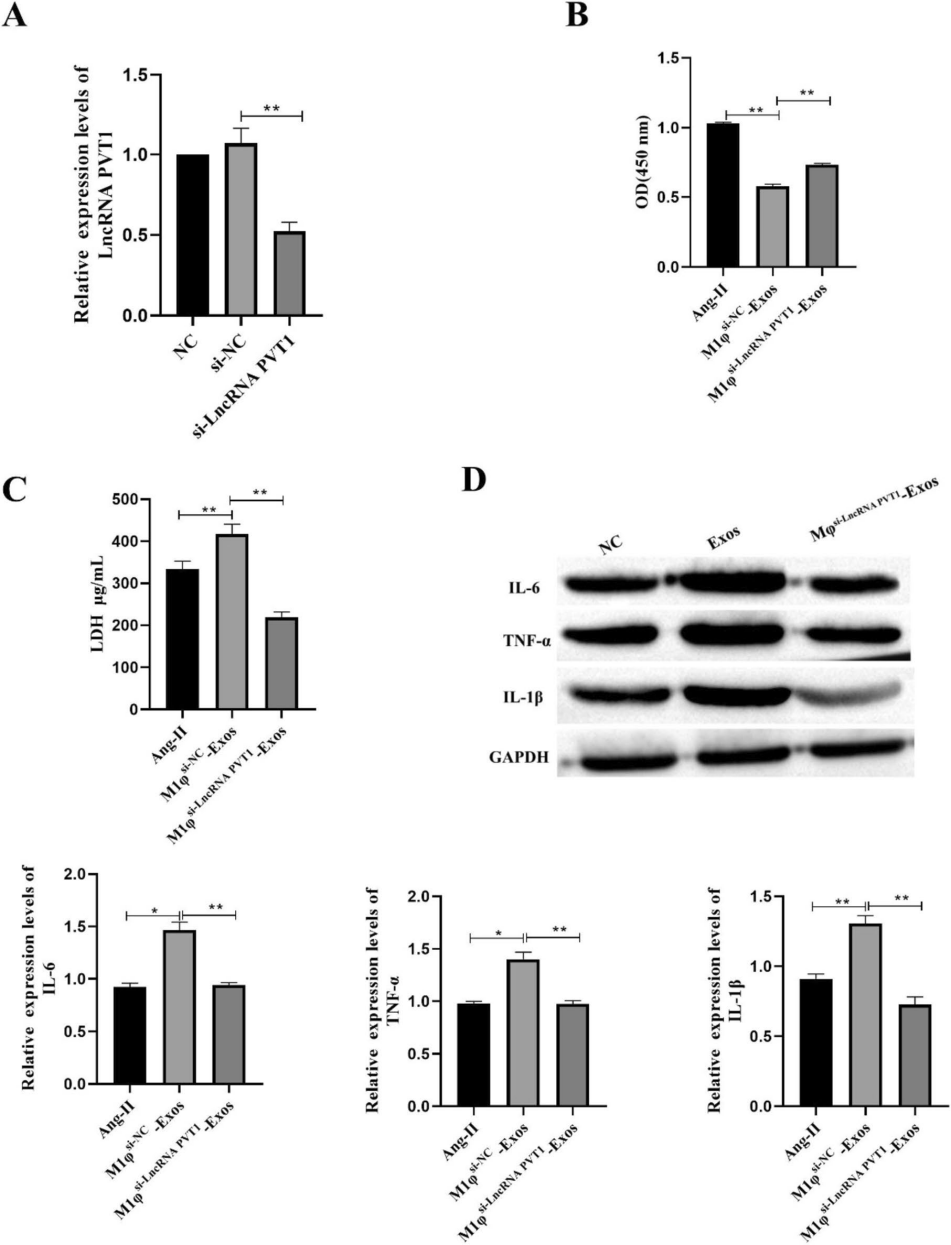
LncRNA PVT1 derived from M1φ-Exos promotes VSMCs inflammation. A The expression of LncRNA PVT1 was detected by RT-qPCR. B CCK-8 was used to detect cell viability; C ELISA was used to detect the concentration of LDH; D Western blot was used to detect inflammatory factors (IL-6, TNF-α and IL-1β). Each group had 3 biological replicates. A one-way ANOVA was performed to compare data between groups.*P < 0.05, **P < 0.01

Previous studies have found that the expression of LncRNA PVT1 in M1φ-Exos increased significantly. In order to explore the effect of M1φ-Exos LncRNA PVT1 on VSMCs inflammation, we transfected si-LncRNA PVT1 into M1φ and detected the transfection efficiency by RT-qPCR. The results showed that the expression of LncRNA PVT1 in M1φ was significantly decreased after transfection of si-LncRNA PVT1 (Fig. 4A). After successful transfection, exosomes (M1φ^si−LncRNA^ PVT1-Exos) were extracted to treat VSMCs. The results showed that compared with M1φsi-NC-Exos, the cell viability of M1φ^si−LncRNA PVT1^-Exos was significantly increased (Fig. 4B), and the concentration of LDH was significantly decreased (Fig. 4C). Western blot detection of IL-6, TNF-α and IL-1β protein expression also found that the expression of IL-6, TNF-α and IL-1β was significantly decreased after M1φ^si−LncRNA PVT1^-Exos treatment (Fig. 4D). The above results indicate that LncRNA PVT1 derived from M1φ-Exos promotes VSMCs inflammation.

### LncRNA PVT1 Derived from M1φ-Exos Promotes VSMCs Pyroptosis

**Fig. 5:**
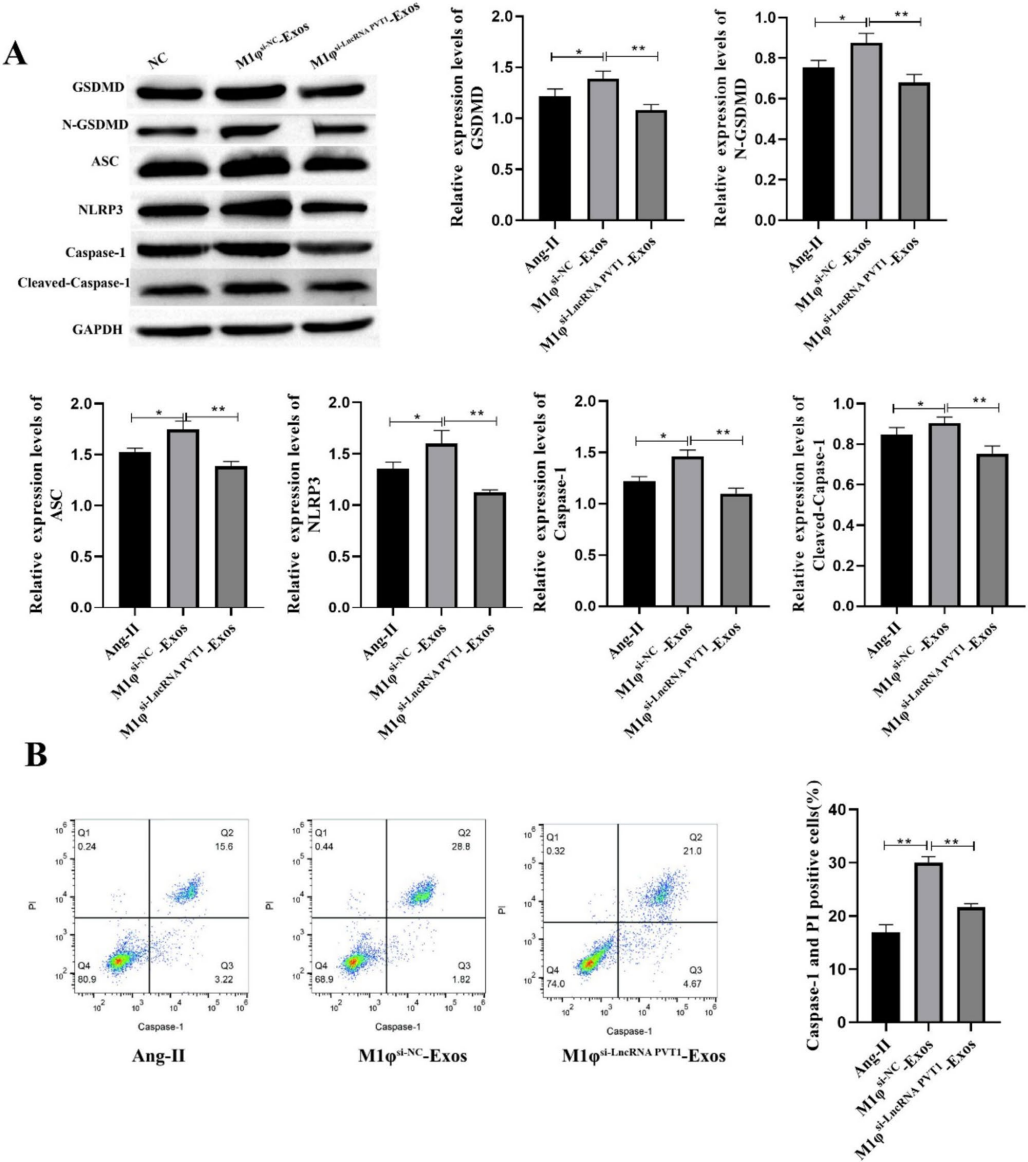
LncRNA PVT1 derived from M1φ-Exos promotes VSMCs pyroptosis. A Western blot was used to detect pyroptosis-related proteins; B pyroptosis was detected by flow cytometry. Each group had 3 biological replicates. A one-way ANOVA was performed to compare data between groups.*P < 0.05, **P < 0.01

Western blot results showed that M1φ^si−LncRNA PVT1^-Exos significantly inhibited the expression of GSDMD, N-GSDMD ASC, NLRP3, Caspase-1 and Cleaved-Caspase-1 compared with M1φ^si−NC^-Exos group (Fig. 5A). Flow cytometry showed that the positive cell rate of Capase-1 was significantly decreased after M1φ^si−LncRNA PVT1^-Exos treatment (Fig. 5B). In summary, LncRNA PVT1 derived from M1φ-Exos can inhibit VSMCs pyroptosis.

### LncRNA PVT1 Targeted Down-Regulated of miRNA-186-5p Expression

**Fig. 6:**
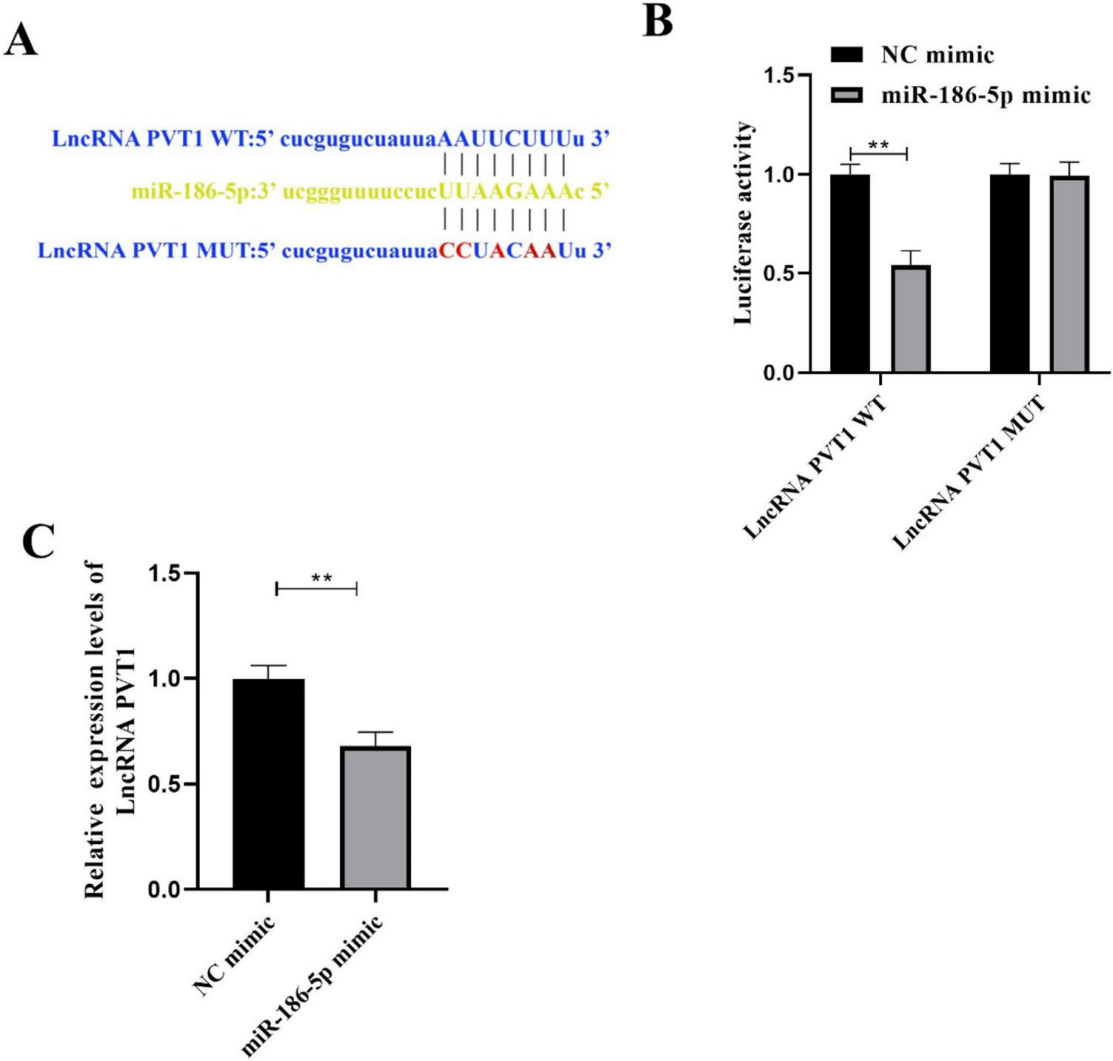
LncRNA PVT1 targeted down-regulated of miRNA-186-5p expression. A StarBase predicted the target binding site of LncRNA PVT1 and miRNA-186-5p. The targeting relationship between LncRNA PVT1 and miRNA-186-5p was verified by B double fluorescein experiment. The expression of LncRNA PVT1 was detected by RT-qPCR. Each group had 3 biological replicates. Student’s *t* test was performed to compare data between groups.*P < 0.05, **P < 0.01

The target binding site of LncRNA PVT1 and miRNA-186-5p was predicted by StarBase (http://starbase.sysu.edu.cn/index.php)) (Fig. 6A). The dual luciferase assay showed that miR-186-5p mimic significantly reduced the luciferase activity of LncRNA PVT1-WT transfected VSMCs cells, but had no effect on the luciferase activity of LncRNA PVT1-MUT (Fig. 6B). At the same time, after VSMCs cells were treated with miR-186 mimic, the expression of LncRNA was significantly decreased (Fig. 6C). In summary, LncRNA PVT1 targeted down-regulated of miRNA-186-5p expression.

### LncRNA PVT1 Derived from M1φ-Exos Promotes Inflammation of VSMCs Through miR-186-5p

**Fig. 7:**
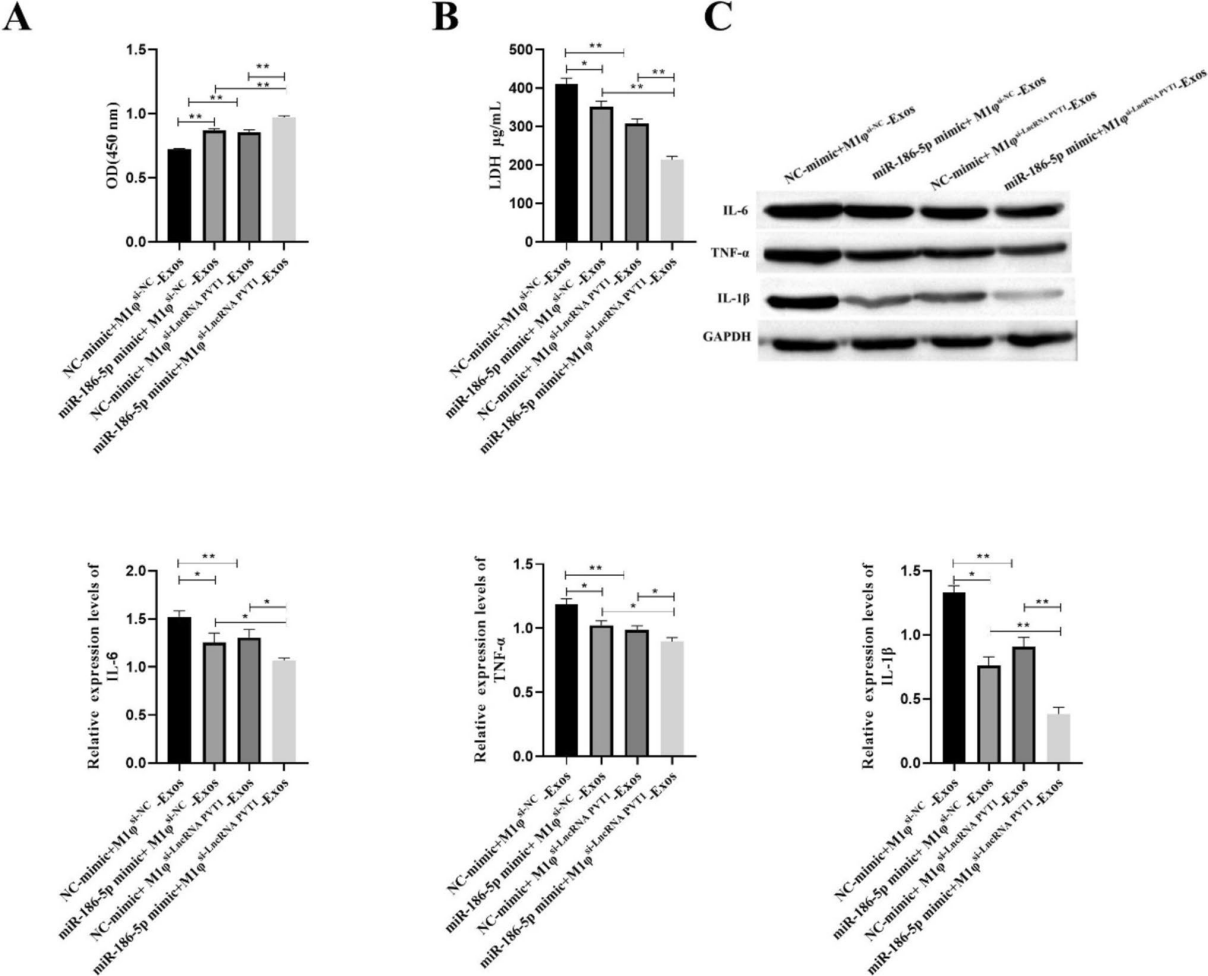
LncRNA PVT1 derived from M1φ-Exos promotes inflammation of VSMCs through miR-186-5p. A CCK-8 was used to detect cell viability; B ELISA was used to detect the concentration of LDH; C Western blot was used to detect inflammatory factors (IL-6, TNF-α and IL-1β). Each group had 3 biological replicates. A one-way ANOVA was performed to compare data between groups.*P < 0.05, **P < 0.01

In order to explore whether M1φ-Exos-derived exosome LncRNA PVT1 can regulate VSMCs inflammation through miR-186-5p, we transfected miR-186-5p into VSMCs. Then, VSMCs were treated with M1φ^si−LncRNA PVT1^-Exos. Compared with the NC-mimic + M1φ^si−NC^-Exos group, the viability of VSMCs cells in the miR-186-5p mimic + M1φ^si−NC^-Exos group and the NC-mimic + M1φ^si−LncRNA PVT1^-Exos group was enhanced (Fig. 7A) and the secretion of LDH was decreased (Fig. 7B). Compared with miR-186-5p mimic + M1φ^si−NC^-Exos group and NC-mimic + M1φ^si−LncRNA PVT1^-Exos group, the cell viability of miR-186-5p mimic + M1φ^si−LncRNA PVT1^-Exos group was further enhanced (Fig. 7A) and the secretion of LDH was further reduced (Fig. 7B). At the same time, the expression of IL-6, TNF-α and IL-1β in miR-186-5p mimic + M1φ^si−NC^-Exos group and NC-mimic + M1φ^si−LncRNA PVT1^-Exos group was significantly decreased. Compared with miR-186-5p mimic + M1φ^si−NC^-Exos group and NC-mimic + M1φ^si−LncRNA PVT1^-Exos group, the expression of IL-6, TNF-α and IL-1β in miR-186-5p mimic + M1φ^si−LncRNA PVT1^-Exos group was significantly decreased (Fig. 7C). The above results indicate that LncRNA PVT1 is derived from M1φ-Exos promotes inflammation of VSMCs through miR-186-5p.

### LncRNA PVT1 Derived from M1φ-Exos Promotes Pyroptosis of VSMCs Through miR-186-5p

**Fig. 8:**
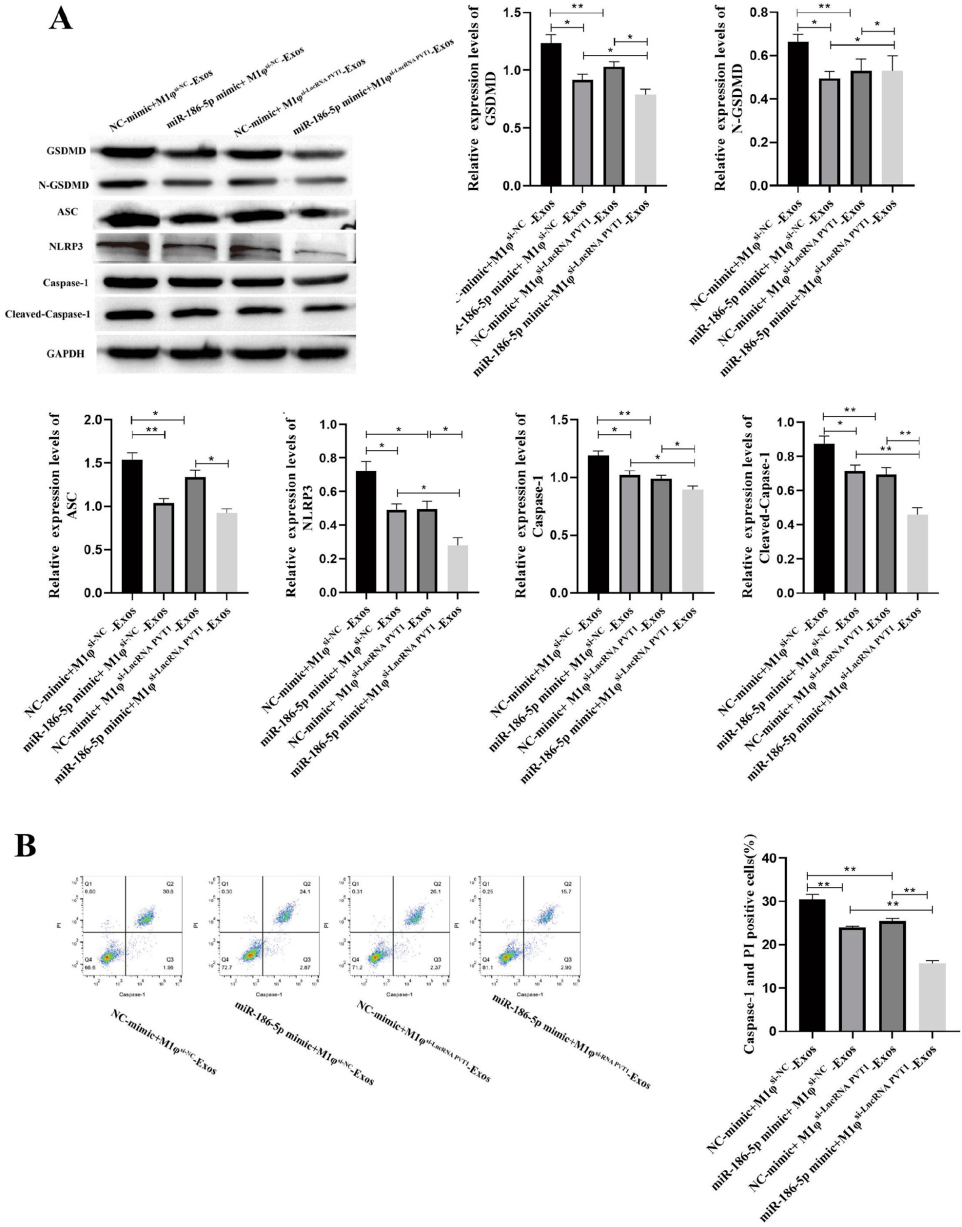
LncRNA PVT1 derived from M1φ-Exos promotes pyroptosis of VSMCs through miR-186-5p. A Western blot was used to detect pyroptosis-related proteins; B pyroptosis was detected by flow cytometry. Each group had 3 biological replicates. A one-way ANOVA was performed to compare data between groups.*P < 0.05, **P < 0.01

The expression of GSDMD, N-GSDMD ASC, NLRP3, Caspase-1 and Cleaved-Caspase-1 was detected by Western blot. The results showed that the expression of GSDMD, N-GSDMD ASC, NLRP3, Caspase-1 and Cleaved-Caspase-1 in miR-186-5p mimic + M1φ^si−NC^-Exos group and NC-mimic + M1φ^si−LncRNA PVT1^-Exos group was significantly lower than that in NC-mimic + M1φ^si−NC^-Exos group. The expression of GSDMD, N-GSDMD ASC, NLRP3, Caspase-1 and Cleaved-Caspase-1 in miR-186-5p mimic + M1φ^si−LncRNA PVT1^-Exos was significantly reduced compared with miR-186-5p mimic + M1φ^si−NC^-Exos group and NC-mimic + M1φ^si−LncRNA PVT1^-Exos group (Fig. 8A). The positive cell rate of Caspase-1 detected by flow cytometry showed that both miR-186-5p mimic and M1φ^si−LncRNA PVT1^ could inhibit the positive cell rate of Caspase-1. The co-treatment of miR-186-5p mimic and M1φ^si−LncRNA PVT1^ further inhibited the positive cell rate of Caspase-1 (Fig. 8B). In summary, LncRNA PVT1 derived from M1φ-Exos promotes pyroptosis of VSMCs through miR-186-5p.

### miR-186-5p Can Target Down-Regulated of HMGB1 Expression

**Fig. 9:**
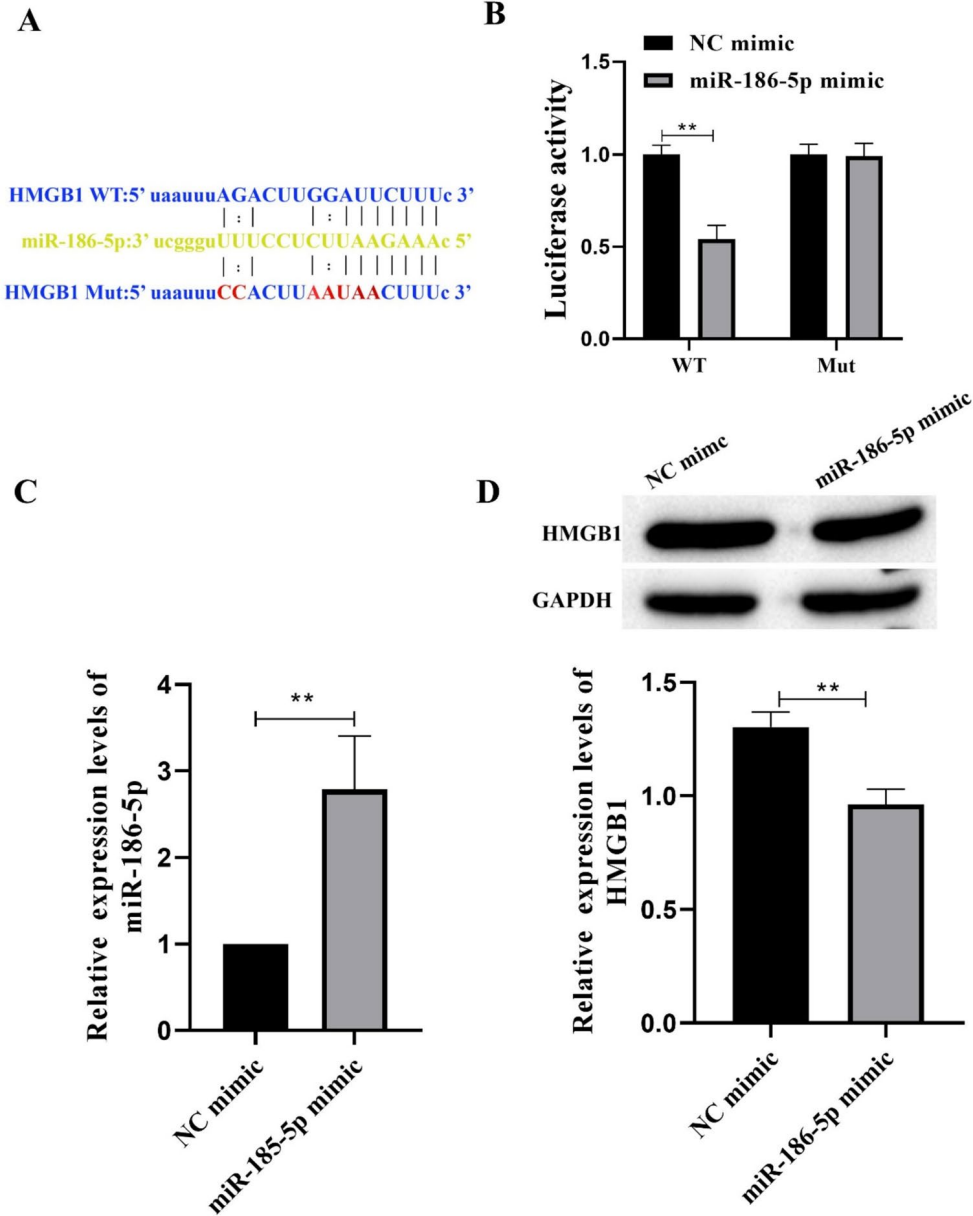
miR-186-5p can target down-regulated of HMGB1 expression. **A** StarBase predicted the target binding site of HMGB1 and miRNA-186-5p. **B** The targeting relationship between HMGB1 and miRNA-186-5p was verified by Dual-Luciferase Reporter Assay. **C** The expression of miRNA-186-5p was detected by RT-qPCR. **D** The expression of HMGB1 was detected byWestern blot. Each group had 3 biological replicates. Student’s *t* test was performed to compare data between groups.*P < 0.05, **P < 0.01

The target binding site of HMGB1 and miRNA-186-5p was predicted by StarBase (http://starbase.sysu.edu.cn/index.php)) (Fig. 9A). The dual luciferase assay showed that miR-186-5p mimic significantly reduced the luciferase activity of HMGB1-WT transfected VSMCs cells, but had no effect on the luciferase activity of HMGB1-MUT (Fig. 9B). The efficiency of miR-186-5p mimic transfected cells was detected by RT-qPCR. The expression of miR-186-5p in VSMCs was significantly increased after miR-186-5p mimic transfection, indicating successful transfection (Fig. 9C).At the same time, after VSMCs cells were treated with miR-186 mimic, the expression of HMGB1 was significantly decreased (Fig. 9D). In summary, miR-186-5p targeted down-regulated of HMGB1 expression.

### miR-186-5p Inhibits Inflammation of VSMCs by Inhibiting HMGB1 Expression

**Fig. 10:**
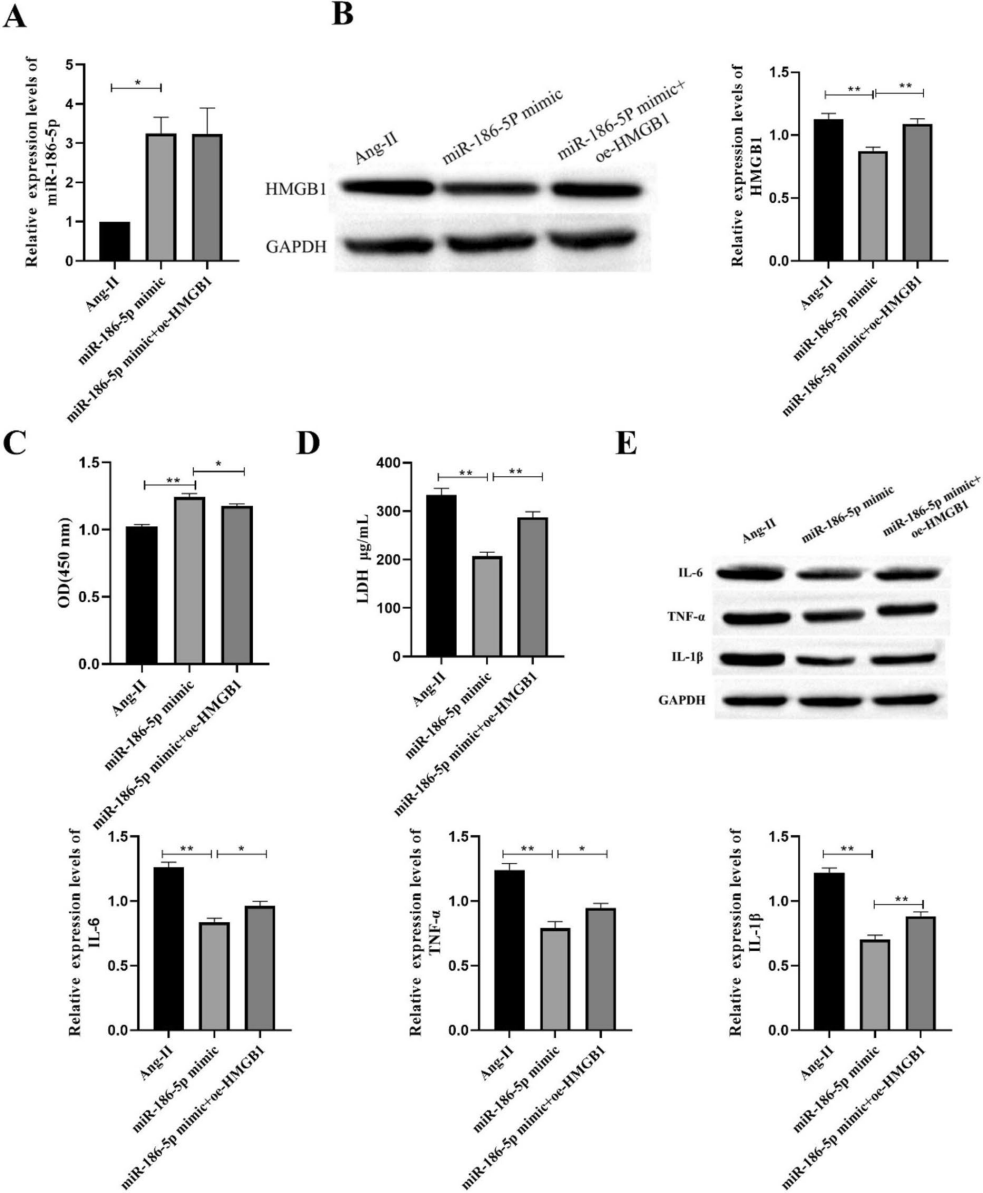
miR-186-5p inhibits inflammation of VSMCs by inhibiting HMGB1 expression. **A** The expression of miRNA-186-5p was detected by RT-qPCR. **B** The expression of HMGB1 was detected byWestern blot. **C** CCK-8 was used to detect cell viability; **D** ELISA was used to detect the concentration of LDH; **E** Western blot was used to detect inflammatory factors (IL-6, TNF-α and IL-1β). Each group had 3 biological replicates. A one-way ANOVA was performed to compare data between groups.*P < 0.05, **P < 0.01

After transfection of miR-186-5p mimic, the expression of miR-186-5p was significantly increased in VSMCs, while oe-HMGB1 treatment had no significant effect on the expression of miR-186-5p (Fig. 10A). At the same time, the expression of HMGB1 was significantly decreased after miR-186-5p mimic transfection, while oe-HMGB1 treatment could significantly increase the expression of HMGB1 (Fig. 10B).miR-186-5p mimic significantly promoted the viability of VSMCs (Fig. 10C) and inhibited the accumulation of LDH (Fig. 10D). oe-HMGB1 can inhibit the effect of miR-186-5p mimic. At the same time, miR-186-5p mimic could significantly inhibit the expression of IL-6, TNF-α and IL-1β (Fig. 10E). oe-HMGB1 could reverse the inhibitory effect of miR-186-5p mimic. The above studies have shown that miR-186-5p inhibits inflammation of VSMCs by inhibiting HMGB1 expression.

### miR-186-5p Inhibits Pyroptosis of VSMCs by Inhibiting HMGB1 Expression

**Fig. 11:**
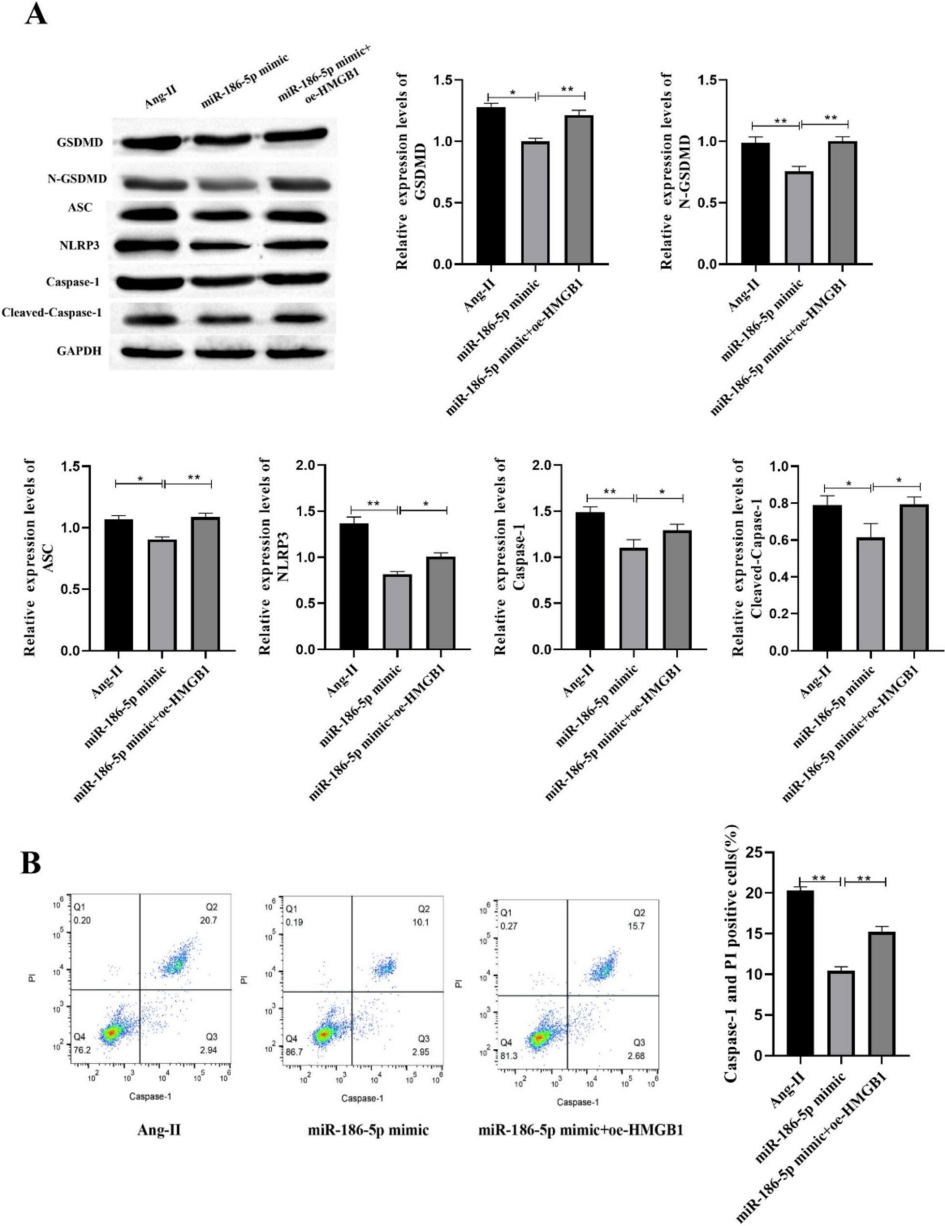
miR-186-5p inhibits pyroptosis of VSMCs by inhibiting HMGB1 expression. A Western blot was used to detect pyroptosis-related proteins; B pyroptosis was detected by flow cytometry. Each group had 3 biological replicates. A one-way ANOVA was performed to compare data between groups. *P < 0.05, **P < 0.01

The expression of GSDMD, N-GSDMD ASC, NLRP3, Caspase-1 and Cleaved-Caspase-1 was detected by Western blot. The results showed that miR-186-5p mimic could significantly inhibit the expression of GSDMD, N-GSDMD ASC, NLRP3, Caspase-1 and Cleaved-Caspase-1, while oe-HMGB1 could inhibit the effect of miR-186-5p mimic (Fig. 11A). The positive cell rate of Capase-1 was detected by flow cytometry. It was found that miR-186-5p mimic could inhibit the positive cell rate of Caspase-1, and oe-HMGB1 reversed the effect of miR-186-5p mimic (Fig. 11B). In summary, miR-186-5p can inhibit VSMCs pyroptosis through HMGB1.

## Discussion

This study has demonstrated that M1φ-derived exosome LncRNA PVT1 is pathogenic in the development of AAA. We found that the expression of LncRNA PVT1 was significantly increased in M1 φ. M1φ^si−LncRNA PVT1^-Exos can inhibit VSMCs inflammation and pyroptosis. At the same time, our experimental results show that there is a targeted regulatory relationship between miR-186-5p and LncRNA PVT1 and HMGB1. It is worth noting that miR-186-5p mimic can further promote the effect of M1φ^si−LncRNA PVT1^-Exos, while oe-HMGB1 can reverse the effect of miR-186-5p mimic. It was proved that miR-186-5p and HMGB1 were key molecules in the formation of AAA. Our study finally demonstrated that M1φ-derived exosome LncRNA PVT1 can inhibit miR-186-5p to regulate HMGB1 to promote VSMCs inflammation and pyroptosis.

Exosomes are involved in the pathophysiological activities of various vascular diseases such as atherosclerosis and vascular calcification. The sources of exosomes are very extensive, including Mφ [22], plasma [23], mesenchymal stem cells [24], etc.Exosomes secreted by these cells affect AAA formation. Mφ plays a key role in the formation and rupture of AAA [25]. Macrophage-derived exosomes aggravate endothelial inflammation by inducing leukocyte adhesion [26]. Nicotine-stimulated exosomes of Mφ accelerate atherosclerosis through miR-21-3p / PTEN-mediated VSMC migration and proliferation [15]. These findings suggest that the exosomes of Mφ may regulate cell–cell interactions in the progression of AAA. This study found that M1φ-derived exosome LncRNA PVT1 was increased in AAA, and M1φ-si-LncRNA PVT1-Exos could significantly reduce inflammation and pyroptosis of VSMC. These results suggest that M1φ-derived secreted LncRNA PVT1 is involved in VSMC inflammation and pyroptosis.

LncRNA acts as a molecular sponge of competitive endogenous molecular RNA to affect the expression of target gene mRNA to exert its biological function [27]. LncRNA H19, as a competitive endogenous RNA, binds to miR-19b-3p to enhance the expression of FTH1 and promote ferroptosis in lung cancer cells [28]. LncRNA XIST binds to miR-34a to regulate MET-PI3K-AKT signaling and affects cell proliferation and growth of thyroid cancer [29]. In this study, we demonstrated that LncRNA PVT1 and HMGB1 can bind to miR-186-5p. In this study, we demonstrated that LncRNA PVT1 can inhibit HMGB1 expression by targeting miR-186-5p.

AAA is a peripheral vascular degenerative disease characterized by chronic inflammation [30]. Inhibition of inflammation-related gene expression is a promising strategy to control the progression of AAA [31]. Injection of monoclonal antibodies against inflammatory factor receptors, such as IL-6 receptors, can inhibit the formation and progression of aneurysms [32]. Itaconic acid ester prevents the formation of abdominal aortic aneurysm by activating Nrf2 to inhibit inflammation [33]. Pyroptosis is a caspase-1-dependent pro-inflammatory cell death characterized by cell swelling and cell membrane dissolution. Inhibition of VSMCs pyroptosis can alleviate the progression of AAA [20]. Increased expression of inflammatory factors in AAA promotes pyroptosis. Previous studies in this study found that M1φ-Exos-derived LncRNA PVT1 can promote VSMCs inflammation and pyroptosis. In addition, after the addition of M1φsi-LncRNA PVT1-Exos and miR-186-5p mimic, VSMCs inflammation and pyroptosis were significantly inhibited, while oe-HMGB1 offset the effect of miR-186-5p mimic. In summary, M1φ-derived exosome LncRNA PVT1 can promote VSMCs inflammation and pyroptosis by miR-186-5p/HMGB1.

**Fig. 12:**
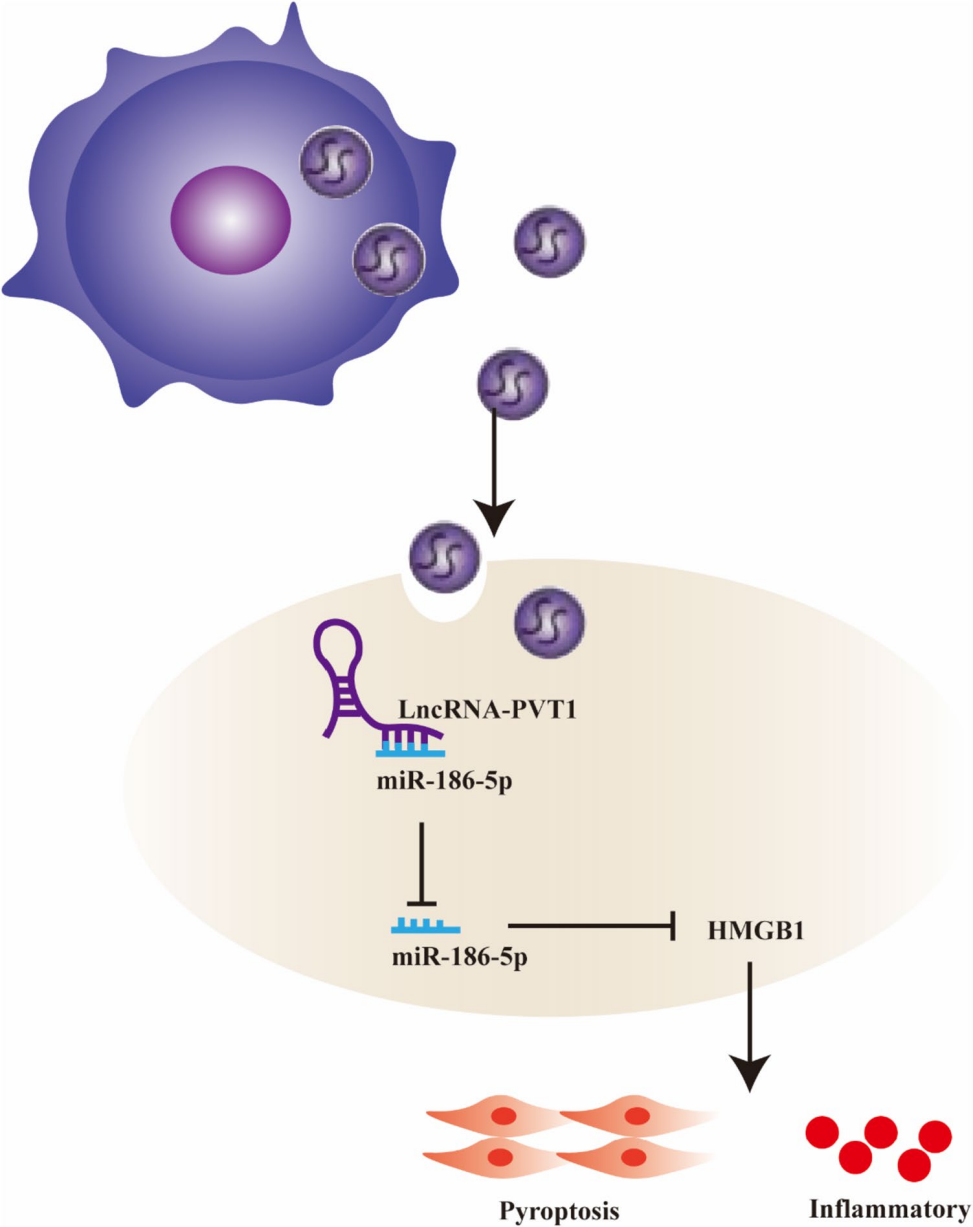
LncRNA PVT1 in exosomes secreted by M1φ can regulate HMGB1 by acting as ceRNA on sponge miR-186-5p, thereby promoting cell inflammatory pyroptosis

In summary, our study determined that M1φ-derived exosome LncRNA PVT1 promotes inflammation and pyroptosis of VSMCs and participates in AAA formation. This effect of promoting HMGB1 is partially induced by the LncRNA PVT1/miR-186-5p ceRNA mechanism (Fig. 12). These results deepen the understanding of lncRNA function in the pathogenesis of AAA. In the future, LncRNA PVT1 has the potential to become a new therapeutic target for the design of AAA therapeutic drugs. However, it should be noted that this study only explored the mechanism of M1φ-derived exosomes in inducing VSMCs inflammation and pyroptosis in cell models. As a result, the implications of these findings are subject to certain limitations. Therefore, future research should aim to verify these findings in vivo models to enhance the credibility of the results.

### Supplementary Information

Below is the link to the electronic supplementary material.

Supplementary file1 (JPG 185 kb)

## Data Availability

All relevant data and materials are available from the authors upon reasonable request.

## References

[1] Hensley SE, Upchurch GR (2022). Repair of abdominal aortic aneurysms: JACC focus seminar, part 1. Journal of the American College of Cardiology.

[2] Kent KC (2014). Clinical practice. Abdominal aortic aneurysms. The New England Journal of Medicine.

[3] Umebayashi R, Uchida HA, Wada J (2018). Abdominal aortic aneurysm in aged population. Aging.

[4] Klink A, Hyafil F, Rudd J, Faries P, Fuster V, Mallat Z, Meilhac O, Mulder WJM, Michel JB, Ramirez F, Storm G, Thompson R, Turnbull IC, Egido J, Martín-Ventura JL, Zaragoza C, Letourneur D, Fayad ZA (2011). Diagnostic and therapeutic strategies for small abdominal aortic aneurysms. Nature Reviews Cardiology.

[5] Lu H, Sun J, Liang W, Chang Z, Rom O, Zhao Y, Zhao G, Xiong W, Wang W, Wang W, Zhu T, Guo Y, Chang L, Garcia-Barrio MT, Zhang J, Chen YE, Fan Y (2020). Cyclodextrin prevents abdominal aortic aneurysm via activation of vascular smooth muscle cell transcription factor EB. Circulation.

[6] Davies LC, Jenkins SJ, Allen JE, Taylor PR (2013). Tissue-resident macrophages. Nature Immunology.

[7] Lu H, Du W, Ren L, Hamblin MH, Becker RC, Chen YE, Fan Y (2021). Vascular smooth muscle cells in aortic aneurysm: From genetics to mechanisms. Journal of the American Heart Association.

[8] Zhao G, Fu Y, Cai Z, Yu F, Gong Z, Dai R, Hu Y, Zeng L, Xu Q, Kong W (2017). Unspliced XBP1 confers VSMC homeostasis and prevents aortic aneurysm formation via FoxO4 interaction. Circulation Research.

[9] Kim W, Lee EJ, Bae IH, Myoung K, Kim ST, Park PJ, Lee KH, Pham AVQ, Ko J, Oh SH, Cho EG (2020). Lactobacillus plantarum-derived extracellular vesicles induce anti-inflammatory M2 macrophage polarization in vitro. Journal of Extracellular Vesicles.

[10] Chinetti-Gbaguidi G, Colin S, Staels B (2015). Macrophage subsets in atherosclerosis. Nature Reviews Cardiology.

[11] Kalluri R, LeBleu VS (2020). The biology, function, and biomedical applications of exosomes. Science.

[12] Wang C, Li Z, Liu Y, Yuan L (2021). Exosomes in atherosclerosis: Performers, bystanders, biomarkers, and therapeutic targets. Theranostics.

[13] Kok VC, Yu CC (2020). Cancer-derived exosomes: Their role in cancer biology and biomarker development. International Journal of Nanomedicine.

[14] Mathivanan S, Ji H, Simpson RJ (2010). Exosomes: Extracellular organelles important in intercellular communication. Journal of Proteomics.

[15] Zhu J, Liu B, Wang Z, Wang D, Ni H, Zhang L, Wang Y (2019). Exosomes from nicotine-stimulated macrophages accelerate atherosclerosis through miR-21-3p/PTEN-mediated VSMC migration and proliferation. Theranostics.

[16] Xu M, Zhou C, Weng J, Chen Z, Zhou Q, Gao J, Shi G, Ke A, Ren N, Sun H, Shen Y (2022). Tumor associated macrophages-derived exosomes facilitate hepatocellular carcinoma malignance by transferring lncMMPA to tumor cells and activating glycolysis pathway. Journal of Experimental & Clinical Cancer Research.

[17] Wang P, Wang H, Huang Q, Peng C, Yao L, Chen H, Qiu Z, Wu Y, Wang L, Chen W (2019). Exosomes from M1-polarized macrophages enhance paclitaxel antitumor activity by activating macrophages-mediated inflammation. Theranostics.

[18] Li X, Li N (2018). LncRNAs on guard. International Immunopharmacology.

[19] Huang Y, Ren L, Li J, Zou H (2021). Long non-coding RNA PVT1/microRNA miR-3127-5p/NCK-associated protein 1-like axis participates in the pathogenesis of abdominal aortic aneurysm by regulating vascular smooth muscle cells. Bioengineered.

[20] Xiong JM, Liu H, Chen J, Zou QQ, Wang YY, Bi GS (2021). Curcumin nicotinate suppresses abdominal aortic aneurysm pyroptosis via lncRNA PVT1/miR-26a/KLF4 axis through regulating the PI3K/AKT signaling pathway. Toxicology Research.

[21] Zhang Z, Zou G, Chen X, Lu W, Liu J, Zhai S, Qiao G (2019). PVT1 knockdown of lncRNA inhibits vascular smooth muscle cell apoptosis and extracellular matrix disruption in a murine abdominal aortic aneurysm model. Molecules and Cells.

[22] Wang Y, Jia L, Xie Y, Cai Z, Liu Z, Shen J, Lu Y, Wang Y, Su S, Ma Y, Xiang M (2019). Involvement of macrophage-derived exosomes in abdominal aortic aneurysms development. Atherosclerosis.

[23] Martinez-Pinna R, Gonzalez de Peredo A, Monsarrat B, Burlet-Schiltz O, Martin-Ventura JL (2014). Label-free quantitative proteomic analysis of human plasma-derived microvesicles to find protein signatures of abdominal aortic aneurysms. Proteomics Clinical Applications.

[24] Zhang Y, Huang X, Sun T, Shi L, Liu B, Hong Y, Fu QL, Zhang Y, Li X (2023). MicroRNA-19b-3p dysfunction of mesenchymal stem cell-derived exosomes from patients with abdominal aortic aneurysm impairs therapeutic efficacy. Journal of Nanobiotechnology.

[25] Davis, F. M., Tsoi, L. C., Melvin, W. J., denDekker, A., Wasikowski, R., Joshi, A. D., Wolf, S., Obi, A. T., Billi, A. C., Xing, X., Audu, C., Moore, B. B., Kunkel, S. L., Daugherty, A., Lu, H. S., Gudjonsson, J. E., & Gallagher, K. A. (2021). Inhibition of macrophage histone demethylase JMJD3 protects against abdominal aortic aneurysms. *The Journal of Experimental Medicine,**218*(6), e20201839. 10.1084/jem.20201839 PMC8008365 33779682

[26] Tang N, Sun B, Gupta A, Rempel H, Pulliam L (2016). Monocyte exosomes induce adhesion molecules and cytokines via activation of NF-κB in endothelial cells. FASEB Journal: Official Publication of the Federation of American Societies for Experimental Biology.

[27] Zhou, C., Yi, C., Yi, Y., Qin, W., Yan, Y., Dong, X., Zhang, X., Huang, Y., Zhang, R., Wei, J., Ali, D. W., Michalak, M., Chen, X. Z., & Tang, J. (2020). LncRNA PVT1 promotes gemcitabine resistance of pancreatic cancer via activating Wnt/β-catenin and autophagy pathway through modulating the miR-619–5p/Pygo2 and miR-619–5p/ATG14 axes. *Molecular Cancer,**19*(1), 118. 10.1186/s12943-020-01237-y PMC7389684 32727463

[28] Zhang, R., Pan, T., Xiang, Y., Zhang, M., Xie, H., Liang, Z., Chen, B., Xu, C., Wang, J., Huang, X., Zhu, Q., Zhao, Z., Gao, Q., Wen, C., Liu, W., Ma, W., Feng, J., Sun, X., Duan, T., Lai-Han Leung, E., Xie, T., Wu, Q., & Sui, X. (2022). Curcumenol triggered ferroptosis in lung cancer cells via lncRNA H19/miR-19b-3p/FTH1 axis. *Bioactive Materials,**13*, 23–36. 10.1016/j.bioactmat.2021.11.013 PMC8843976 35224289

[29] Liu H, Deng H, Zhao Y, Li C, Liang Y (2018). LncRNA XIST/miR-34a axis modulates the cell proliferation and tumor growth of thyroid cancer through MET-PI3K-AKT signaling. Journal of Experimental & Clinical Cancer Research.

[30] IsIsoda, K., Akita, K., Kitamura, K., Sato-Okabayashi, Y., Kadoguchi, T., Isobe, S., Ohtomo, F., Sano, M., Shimada, K., Iwakura, Y., & Daida, H. (2018). Inhibition of interleukin-1 suppresses angiotensin II-induced aortic inflammation and aneurysm formation. *International Journal of Cardiology,**270*, 221–227. 10.1016/j.ijcard.2018.05.072 29884291

[31] Yuan Z, Lu Y, Wei J, Wu J, Yang J, Cai Z (2020). Abdominal aortic aneurysm: Roles of inflammatory cells. Frontiers in Immunology.

[32] Sun, Y., Zhong, L., He, X., Wang, S., Lai, Y., Wu, W., Song, H., Chen, Y., Yang, Y., Liao, W., Liao, Y., & Bin J. (2019). LncRNA H19 promotes vascular inflammation and abdominal aortic aneurysm formation by functioning as a competing endogenous RNA. *Journal of Molecular and Cellular Cardiology,**131*, 66–81. 10.1016/j.yjmcc.2019.04.004 30991034

[33] Song, H., Xu T., Feng, X., Lai, Y., Yang, Y., Zheng, H., He, X., Wei, G., Liao W., Liao, Y., Zhong, L., & Bin, J. (2020). Itaconate prevents abdominal aortic aneurysm formation through inhibiting inflammation via activation of Nrf2. *eBioMedicine,**57*, 102832. 10.1016/j.ebiom.2020.102832 PMC7322255 32574955

