# Supplementary figures and images for: M1 Macrophage-Derived Exosome LncRNA PVT1 Promotes Inflammation and Pyroptosis of Vascular Smooth Muscle Cells in Abdominal Aortic Aneurysm by Inhibiting miR-186-5p and Regulating HMGB1
Source: Cardiovasc Toxicol. 2024 Mar 7;24(3):302–20. doi: 10.1007/s12012-024-09838-5 (PMC10937795; doi:10.1007/s12012-024-09838-5)

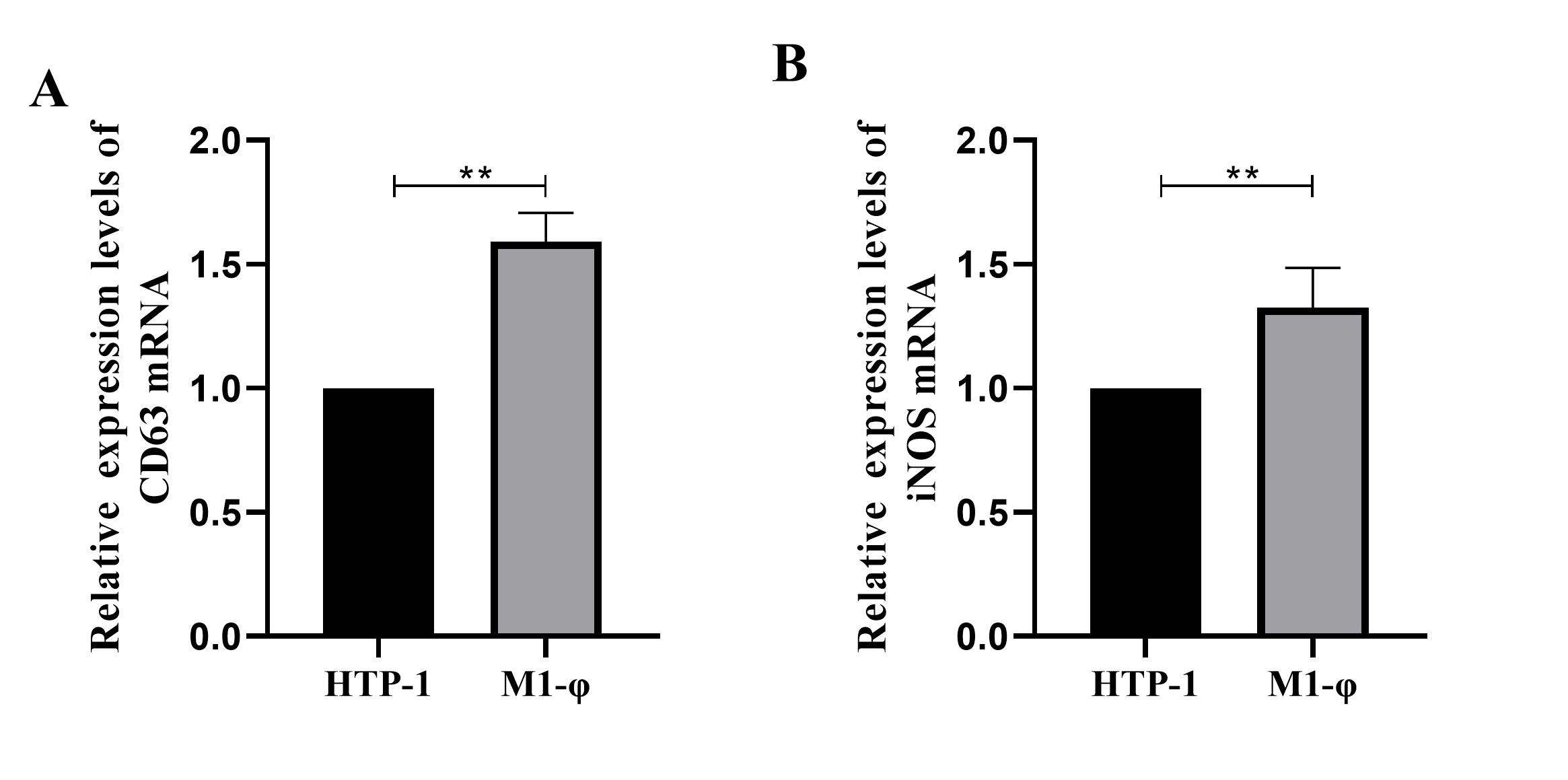

Supplement: Supplementary file 1 — Supplementary file1 (JPG 185 kb) [file 12012_2024_9838_MOESM1_ESM.jpg]
